# Identification of a new family of putative PD-(D/E)XK nucleases with unusual phylogenomic distribution and a new type of the active site

**DOI:** 10.1186/1471-2164-6-21

**Published:** 2005-02-18

**Authors:** Marcin Feder, Janusz M Bujnicki

**Affiliations:** 1 Laboratory of Bioinformatics and Protein Engineering, International Institute of Molecular and Cell Biology, Trojdena 4, 02-109 Warsaw, Poland

## Abstract

**Background:**

Prediction of structure and function for uncharacterized protein families by identification of evolutionary links to characterized families and known structures is one of the cornerstones of genomics. Theoretical assignment of three-dimensional folds and prediction of protein function even at a very general level can facilitate the experimental determination of the molecular mechanism of action and the role that members of a given protein family fulfill in the cell. Here, we predict the three-dimensional fold and study the phylogenomic distribution of members of a large family of uncharacterized proteins classified in the Clusters of Orthologous Groups database as COG4636.

**Results:**

Using protein fold-recognition we found that members of COG4636 are remotely related to Holliday junction resolvases and other nucleases from the PD-(D/E)XK superfamily. Structure modeling and sequence analyses suggest that most members of COG4636 exhibit a new, unusual variant of the putative active site, in which the catalytic Lys residue migrated in the sequence, but retained similar spatial position with respect to other functionally important residues. Sequence analyses revealed that members of COG4636 and their homologs are found mainly in Cyanobacteria, but also in other bacterial phyla. They undergo horizontal transfer and extensive proliferation in the colonized genomes; for instance in *Gloeobacter violaceus *PCC 7421 they comprise over 2% of all protein-encoding genes. Thus, members of COG4636 appear to be a new type of selfish genetic elements, which may fulfill an important role in the genome dynamics of Cyanobacteria and other species they invaded. Our analyses provide a platform for experimental determination of the molecular and cellular function of members of this large protein family.

**Conclusion:**

After submission of this manuscript, a crystal structure of one of the COG4636 members was released in the Protein Data Bank (code 1wdj; Idaka, M., Wada, T., Murayama, K., Terada, T., Kuramitsu, S., Shirouzu, M., Yokoyama, S.: Crystal structure of Tt1808 from *Thermus thermophilus *Hb8, *to be published*). Our analysis of the Tt1808 structure reveals that we correctly predicted all functionally important features of the COG4636 family, including the membership in the PD-(D/E)xK superfamily of nucleases, the three-dimensional fold, the putative catalytic residues, and the unusual configuration of the active site.

## Background

The PD-(D/E)XK domain is ubiquitously found in enzymes involved in metabolism of nucleic acids, mostly in nucleases with diverse biological functions. The first structurally characterized members of the PD-(D/E)XK superfamily were restriction enzymes (REases) (reviews: [1,2]). Crystallographic studies revealed that this superfamily groups together many nucleases with different cellular functions, including: phage λ exonuclease [3], bacterial enzymes exerting ssDNA nicking in the context of methyl-directed and very-short-patch DNA repair: MutH [4] and Vsr [5], Tn7 transposase TnsA [6], a family of archaeal Holliday junction resolvases (Hjc and Hje) from different species of Archaea [7-9], a Holliday junction resolvase (endonuclease I) from phage T7 [10], and an archaeal XPF/Rad1/Mus81 family nuclease that cleaves branched structures generated during DNA repair, replication, and recombination [11].

All members of the PD-(D/E)XK superfamily share a common structural core, comprising a mixed β-sheet of 4 or 5 strands flanked on both sides by α-helices [1,2,12]. These secondary structures are often embedded in very different peripheral elements, which sometimes constitute the majority of the protein. The common β-sheet serves as a scaffold for a weakly conserved active site, typically comprising two or three acidic residues (Asp or Glu) and one Lys residue, which together form the hallmark bipartite catalytic motif (P)D...X_n_...(D/E)XK (where X is any amino acid). The Lys residue serves to position a water molecule for an in-line attack on the scissile phosphodiester bond, while the carboxylate residues coordinate a Mg^2+ ^ion, which acts as a cofactor. Despite the wealth of structural and biochemical data, obtained mainly for REases (summarized in a collection of reviews: [13]), there is still controversy over the exact catalytic mechanism and the number of metal ions required (1, 2, or 3) by PD-(D/E)XK nucleases [14,15]. Moreover, it was found that some members of the PD-(D/E)XK superfamily developed different variants of the active site. In Vsr and its homologs, the (D/E)XK half-motif was replaced by "FxH" and an additional, unique catalytic His residue appeared in another part of the common three-dimensional fold [5]. In some REases, the acidic residue from the (D/E)XK half-motif was found to have "migrated" to another region of the polypeptide in a way that the position of the carboxylate group in the active site is generally maintained as in the "orthodox" members of the PD-(D/E)XK superfamily, despite the side chain is attached to another place in the backbone [16-19]. In a few enzymes, the conserved Lys was found to be replaced by a Glu, Gln, or Asn residue [20-22].

Crystallographic analyses have also revealed the PD-(D/E)XK fold in proteins that do not function as deoxyribonucleases at all and exhibit no conservation of the active site with the above-mentioned enzymes. The structure of the C-terminal catalytic domain of tRNA splicing endoribonuclease (RNase) EndA is identical to the minimal core of the PD-(D/E)XK fold [23], yet this protein lacks the Mg^2+ ^binding site common to its cousins that cleave phosphodiester bonds in DNA. Remarkably, on the opposite side of the common fold, EndA developed a different active site, whose geometric configuration is very similar to that of a His-Tyr-Lys triad in structurally unrelated RNase A [24]. Finally, the N-terminal domain (NTD) of the RPB5 subunit of RNA polymerase from *Saccharomyces cerevisiae *exhibits perfect conservation of the restriction enzyme-like structure, but lacks any catalytic residues – it is postulated that it functions as a nucleic acid binding domain devoid of any catalytic activity [25].

The divergence exhibited by the members of the PD-(D/E)XK superfamily is remarkable. Even enzymes with very similar biological functions, such as REases that recognize and cleave the same substrate, can exhibit little or no significant sequence similarity. Thus, most of the afore-mentioned enzymes were considered unrelated until the corresponding crystal structures were solved. Only in a few cases the membership in the PD-(D/E)XK superfamily was successfully predicted using bioinformatics (in some cases backed up by mutagenesis of hypothetical catalytic residues) before the actual structures were determined [26-29]. The catalogue of members of the PD-(D/E)XK superfamily is therefore far from being complete and it is expected that new lineages will be discovered as new sequences appear in the databases. Here, we predict that a large uncharacterized protein family with an unusual phylogenetic distribution is likely to represent a new branch of PD-(D/E)XK nucleases.

## Results

### Sequence analysis of COG4636 reveals remote similarity to PD-(D/E)XK nucleases

**Figure 1:**
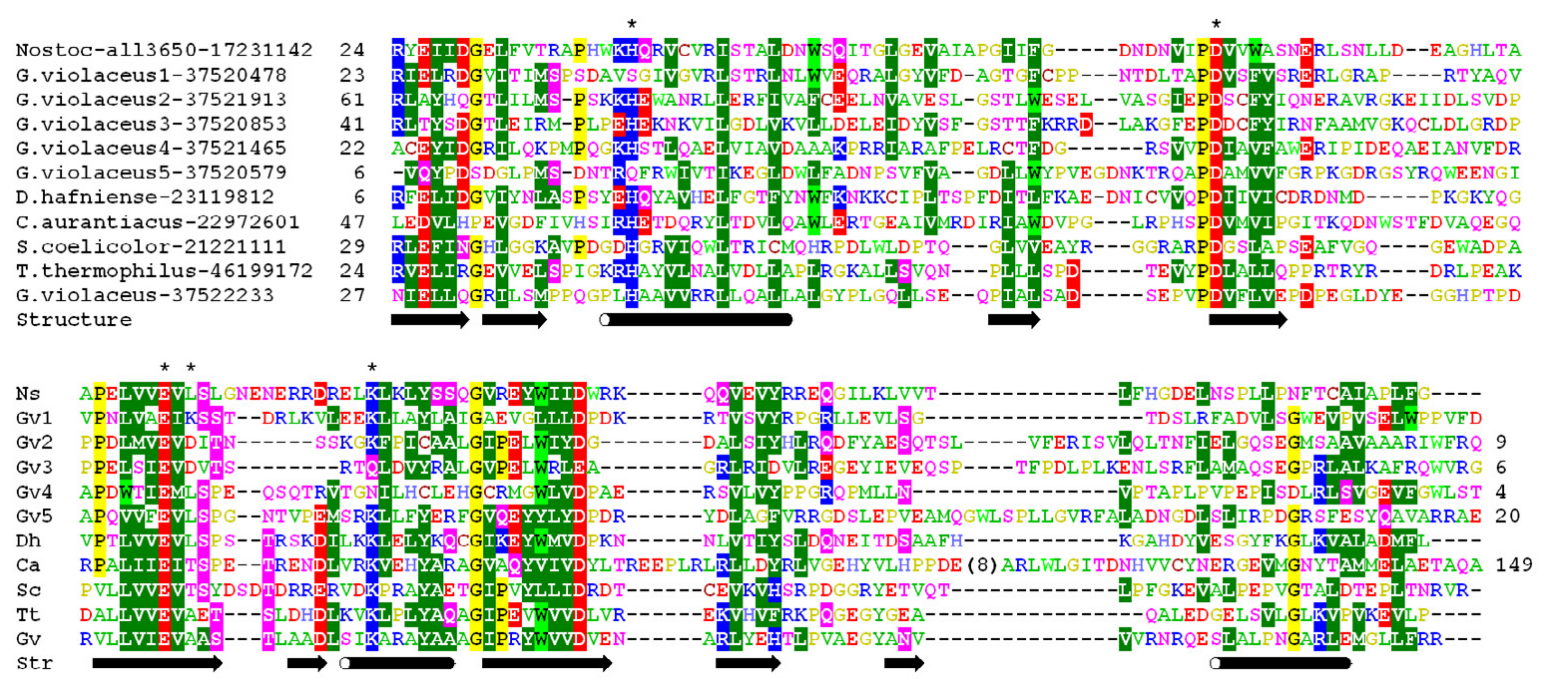
**Multiple sequence alignment of selected representatives of the extended COG4636+ family. **The selection of representative sequences includes the modeled protein from Nostoc (motif H-PD-EXX-K, members from *G. violaceus *with different order of putative catalytic residues (Gv1: H-PD-EXK; Gv2: S-PD-EXD-K; Gv3: H-PD-EXD; Gv4: H-PD-EXX-N; Gv5: Q-PD-EXX-K), and members of mono-phyletic clusters from *D. hafniense*, *C. aurantiacus*, *S. coelicolor*, *T. thermophilus*, and *G. violaceus*). The positions of putative catalytic residues are labeled with "*". The variable termini, which could not be confidently aligned, are not shown; the number of omitted residues is indicated. A complete alignment of full-length sequences is available for download from . Amino acids are colored according to the physico-chemical properties of their side-chains (negatively charged: red, positively charged: blue, polar: magenta, hydrophobic: green). Conserved residues are highlighted. Elements of predicted secondary structure (helices and strands) are indicated by tubes and arrows, respectively.

In the course of analyses of proteins with unknown structures, we came across a family of sequences grouped together in the Clusters of Orthologous Groups (COG) database [30] as COG4636 and annotated as "uncharacterized protein conserved in Cyanobacteria". Analyses of cross-references to other databases revealed no functional information about any member of this family. Nonetheless, preliminary analysis of sequence conservation combined with secondary structure prediction revealed a characteristic pattern of α-helices and β-strands associated with conserved carboxylate residues (review: [31]), which suggested that members of COG4636 may belong to the PD-(D/E)XK superfamily (Figure 1). The multiple sequence alignment revealed nearly perfect conservation of a "PD" half-motif, but only partial conservation of the "(D/E)XK" half-motif. Specifically, instead of the Lys residue most members of COG4636 possessed a hydrophobic amino-acid, such as Leu or Val. This suggested that the apparent similarity to the pattern of catalytic residues typical for the PD-(D/E)XK superfamily may be either spurious or indicate a new family of enzymes with an active site devoid of the otherwise conserved residue. We searched for homologs of the analyzed family, beyond sequences from complete genomes grouped together in COG4636, by carrying PSI-BLAST searches of the nr database. Altogether, we collected 435 sequences with significant similarity to COG4636, which will be hereafter referred to as "COG4636+". No statistically significant sequence similarity was detected to any protein with an experimentally determined function.

In order to test the hypothesis of the evolutionary connection between COG4636 and the PD-(D/E)XK superfamily we carried out the fold-recognition analysis, which allows to predict the three-dimensional fold of the target protein by matching its sequence with the available protein structures and assessing the sequence-structure compatibility using a combination of criteria, such as sequence similarity, match of secondary structure elements, compatibility of residue-residue contacts, etc. (review: [32]). Sequences of individual members of COG4636 were therefore submitted to the GeneSilico protein fold-recognition metaserver [33]. Disappointingly, no methods reported statistically significant matches between these sequences and proteins with known structures. Only a few threading methods that explicitly use the structural information from the templates (FUGUE, INBGU, mGenTHREADER, SAM-T02, and 3DPSSM) reported, in some cases, matches to structures of PD-(D/E)XK nucleases, but never at the first position of the ranking. However, in the course of CASP-5 protein structure prediction contest we found that the fold-recognition operation for strongly diverged proteins can be greatly improved by limiting the analysis to the conserved core, i.e. omission of strongly diverged regions and non-conserved insertions, as well as using a refined multiple sequence alignment rather than allowing the servers to build their own sequence profiles from unrefined PSI-BLAST results [34]. Thus, we modified the multiple sequence alignment of the COG4636+ family by removing strongly diverged termini that could not be reliably aligned, and submitted to the meta-server only the core section, comprising ca. 110 aa. This time, as expected, fold-recognition analysis of a well-defined protein core gave unambiguous results: mGenTHREADER, SPARKS, and FUGUE reported structures of Holliday junction resolvases Hjc and Hje, members of the PD-(D/E)XK fold [7-9], at the first positions of their rankings, with significant scores (0.45, -2.08, and 3.46, respectively). Results obtained from the primary servers have been supported by the consensus server Pcons [35], which reported the Hjc and Hje enzymes at the first four position of its ranking, with scores 1.38-1.20, compared to the insignificant score 0.61 for the subsequent fold in the ranking.

### Modeling and model-based identification of a putative active site

**Figure 2:**
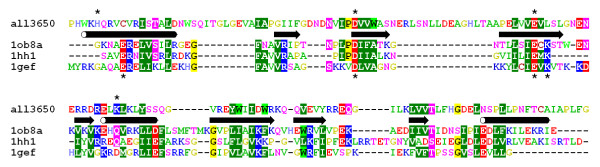
**Fold-recognition alignment between all3650 and structures of Hjc and Hje. **Amino acids are colored according to the physico-chemical properties of their side-chains. Conserved residues are highlighted. Secondary structure elements experimentally identified in Hjc and Hje and predicted for all3650 are shown between the target and the template sequences. Known and predicted catalytic residues are indicated by "*" (above the alignment for the target, below the alignment for the templates).

**Figure 3:**
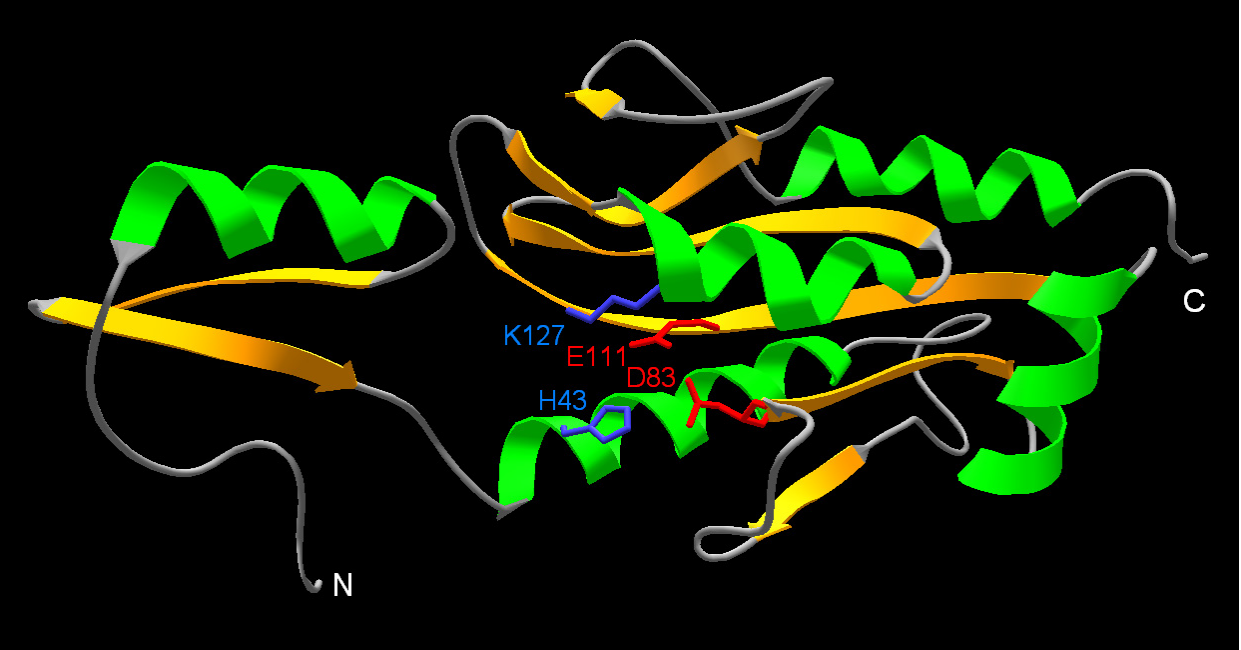
**Homology model of all3650. **Helices and strands are shown in green and yellow, respectively. The predicted catalytic residues are shown in the wireframe representation and labeled. The termini are indicated.

In order to identify the putative active site of newly predicted members of the PD-(D/E)XK superfamily, we modeled the structure of one of the COG4636+ members, whose sequence was close to the consensus calculated for the whole family (hypothetical protein all3650 from *Nostoc *sp. PCC 7120, GI: 17231142) and used it as a platform to study the three-dimensional arrangement of conserved residues. A homology model of all3650 was constructed using the "FRankenstein's Monster" approach (see Methods and ref. [34]), starting with the unrefined alignments between the consensus sequence and the structures of Hjc and Hje enzymes (1gef, 1hh1, and 1ob8) reported by threading methods. Initially, the model of the protein core was constructed by iterating the homology modeling procedure, evaluation of the sequence-structure fit by VERIFY3D, merging of fragments with best scores, and local realignment in poorly scored regions. Local realignments were constrained to maintain the overlap between the secondary structure elements found in the template structures, and those predicted for the target. This procedure was stopped when the regions in the protein core (helices and strands) obtained acceptable VERIFY3D score (>0.3) or their score could not be improved by any manipulations, while the average VERIFY3D score for the whole model could not be improved. The final alignment between all3650 and the three structures used as templates is shown in Figure 2. The final model of the core, comprising residues 39–188, obtained a poor average VERIFY3D score of 0.13 due to low scores in the variable loops that could not be modeled with confidence. However, the secondary structure elements (with the exception of the C-terminal helix), obtained an acceptable average score of 0.37. It is important to note that all catalytic residues of the PD-(D/E)XK fold are found in the stable regions of regular secondary structure rather than in loops [36]. The variable N-terminus, which could not be modeled because of the strong divergence and the lack of appropriate template structures, was added "de novo" using the fragment insertion method ROSETTA [37]. The coordinates of the final, full-length model (Figure 3) are available as supplementary material [see Additional file 1] and on-line at 

**Figure 4:**
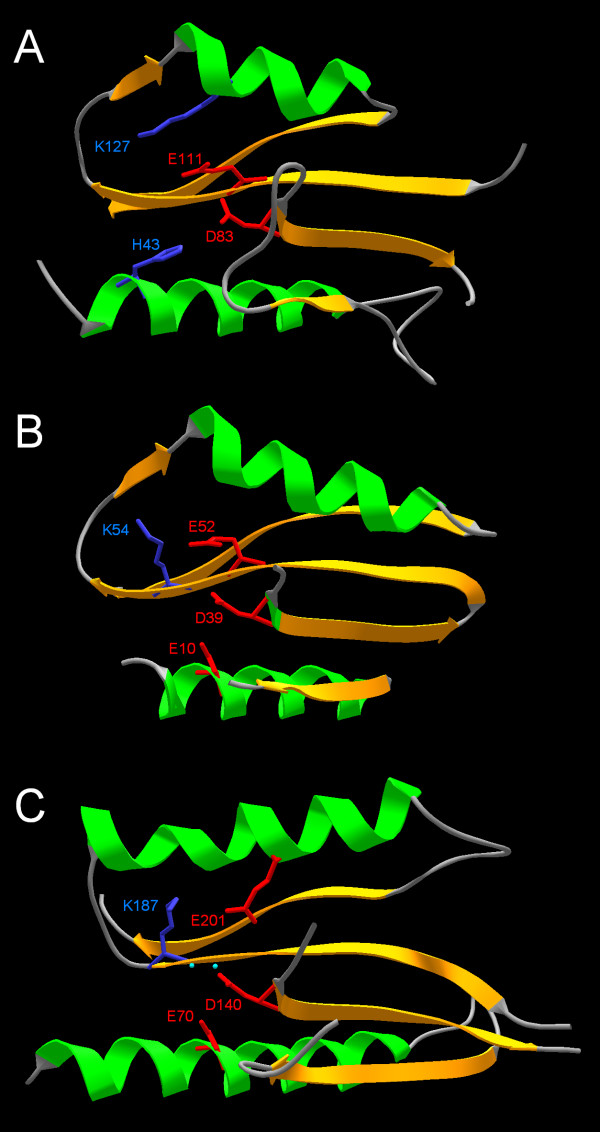
**Spatial conservation of the PD-(D/E)XK active site in all3650, Hjc, and NgoMVI. **A) The predicted structure of all360 is shown in the same orientation as the crystal structures of the bona fide PD-(D/E)XK nucleases: B) Holliday junction resolvase Hje (1ob8 in PDB [9]) and C) REase NgoMIV (1fiu in PDB [78] to illustrate the spatial conservation of side-chains in the active site (the carboxylate residues in red and the Lys residue in blue), despite the lack of their conservation in the PD-EXX-K, PD-DXK, and PD-XXK-E variants of the sequence motif. Only the common core is shown, terminal regions and insertions have been omitted for clarity of the presentation.

The model of all3650 reveals a typical PD-(D/E)XK nuclease-like spatial arrangement of one Lys ε-amino group (from the residue K127) and two carboxylate groups (from residues D83 and E111) (Figure 4). The modeled structure suggest also an additional highly conserved His residue (H43) that could be a part of the metal ion-binding site or be involved in substrate-binding. Strikingly, in all3650 as well as in the great majority of sequences from the COG4636+ family, the conserved Lys (K127 in all3650) is found not in the common position in the same β-strand as the conserved Glu residue (E111 in all3650), but in a spatially adjacent α-helix. Thus, the predicted active site is formed by a "PD-EXX-K" sequence motif. This "migration" of the presumptive catalytic Lys residue and retention of the original position of the spatially adjacent carboxylate in COG4636+ members resembles the situation reported for a number of restriction enzymes such that as Cfr10I, NgoMIV, Ecl18kI, SsoII, and PspGI [16,18,19,38]. In the latter enzymes, however, it is the carboxylate that is relocated and the original position of the Lys residue is retained, such the active site is formed by a "PD-XXK-E" sequence motif (Figure 4).

Inspection of the multiple sequence alignment reveals that only two carboxylates (corresponding to D83 and E111 in all3650) are practically invariant in the COG4636+ family, while all the others undergo various substitutions (Figure 1). In a small group of sequences (represented by a hypothetical protein gll0909 from *G. violaceus*, GI: 37520478) the Lys residue is present both at the "classical" and alternative position, thereby forming a "PD-EXK-K" variant of the active site. This arrangement resembles a putative evolutionary intermediate between the "classical" active site and the newly discovered rearranged variant. In another lineage of the COG4636+ family, an Asp residue appears in the position normally occupied by Lys in the C-terminal half-motif. Some of the members of this lineage (exemplified by glr2344 from *G. violaceus*, GI: 37521913) exhibit therefore the "PD-EXD-K" motif, but the majority (exemplified by hypothetical protein glr1284 from *G. violaceus*, GI: 37520853) lack the Lys residue and exhibit only the "PD-EXD" variant. In another lineage (represented by gll1896 from *G. violaceus*, GI: 37521465) the Lys residue is replaced by Asn to form the "PD-EXX-N" variant of the predicted active site. The conserved His residue (H43 in all3650) is present in most members of the COG4636+ family, with the exception of a small lineage of closely related proteins (represented by gll1896 from *G. violaceus*, GI: 37520579) in which it is substituted by Gln, and a larger group of more diversified sequences, in which it is substituted by Thr or Ser. Most members of the latter group possess a Lys or Arg residue in the "catalytic" position and hence exhibit "PD-EXK-K" (see above) or "PD-EXR-K" variants of the active site. It will be very interesting to determine experimentally, which of those residues in different configurations are involved in catalysis, and which are only auxiliary. In particular, it would be interesting to find if both or either of the Lys residues present in the potential "intermediate" versions of the active site are required for catalysis.

### Phylogenomic analysis of the COG4636+ family

Sequence searches of the nr database at the NCBI revealed that the great majority of members of the COG4636+ family (382 of total 435) originate from Cyanobacteria; of these, 84% were found in just 6 genomes (*G. violaceus *PCC 7421, *Nostoc punctiforme *PCC 73102, *Crocosphaera watsonii *WH 8501, *Nostoc *sp. PCC 7120, *Anabaena variabilis *ATCC 29413, *Synechocystis *sp. PCC 6803). It is astonishing that members of COG4636+ represent over 2% of all protein-encoding genes of *G. violaceus *PCC 7421 (95 of 4430 total [39]), other completely sequenced genomes of Cyanobacteria are completely devoid of them or encode only 1 or 2 sequences from this family. We were not able to identify any members of the COG4636+ family in the sequences derived from seawater samples collected from the Sargasso Sea [40] and deposited in the "environmental samples" database at the NCBI. Since the prevalent Cyanobacteria found in the Sargasso Sea are *Synechococcus *and *Prochlorococcus*, the lack of COG4636+ members in the environmental samples is in good agreement with the paucity of these genes in the fully sequenced genomes of these species.

In order to reconstruct the evolutionary history of the COG4636+ family, we calculated the phylogenetic tree, based on the same reliable section of the multiple sequence alignment that was used for protein structure prediction (see Methods). Unfortunately, in all trees obtained with different methods and parameters, the majority of deep branches received very low bootstrap support (data not shown), hence the relationships within the whole family must be regarded as unresolved. We were able, however, to identify a number of branches with bootstrap support >90%. Many of such branches comprise members from one species only. This situation is characteristic for sequences found in a few non-Cyanobacterial species; for instance 8 sequences from *D. hafniense *DCB-2 (Firmicutes), 7 sequences from *C. aurantiacus *(Chloroflexales), and 6 from *S. coelicolor *(Actinobacteria) each form a separate species branch on the phylogenetic tree, while 14 sequences from *T. thermophilus *HB27 (Deinococcus-Thermus lineage) form three separate branches. Several monophyletic groups of closely related sequences are also observed in *G. violaceus *(e.g. a sub-family comprising 7 sequences with GI numbers: 37522824, 37520777, 37522646, 37521452, 37522233, 37520151, 37522558). There is also one branch comprising 6 closely related sequences in *C. watsonii*, GI numbers: 45527153, 45527776, 45524526, 45527777, 45527775, 45527774). Other statistically significant branches, however, comprise members from different species, suggesting that they were either formed prior to speciation or that their members were transmitted horizontally between different genomes of already existing species.

**Figure 5:**
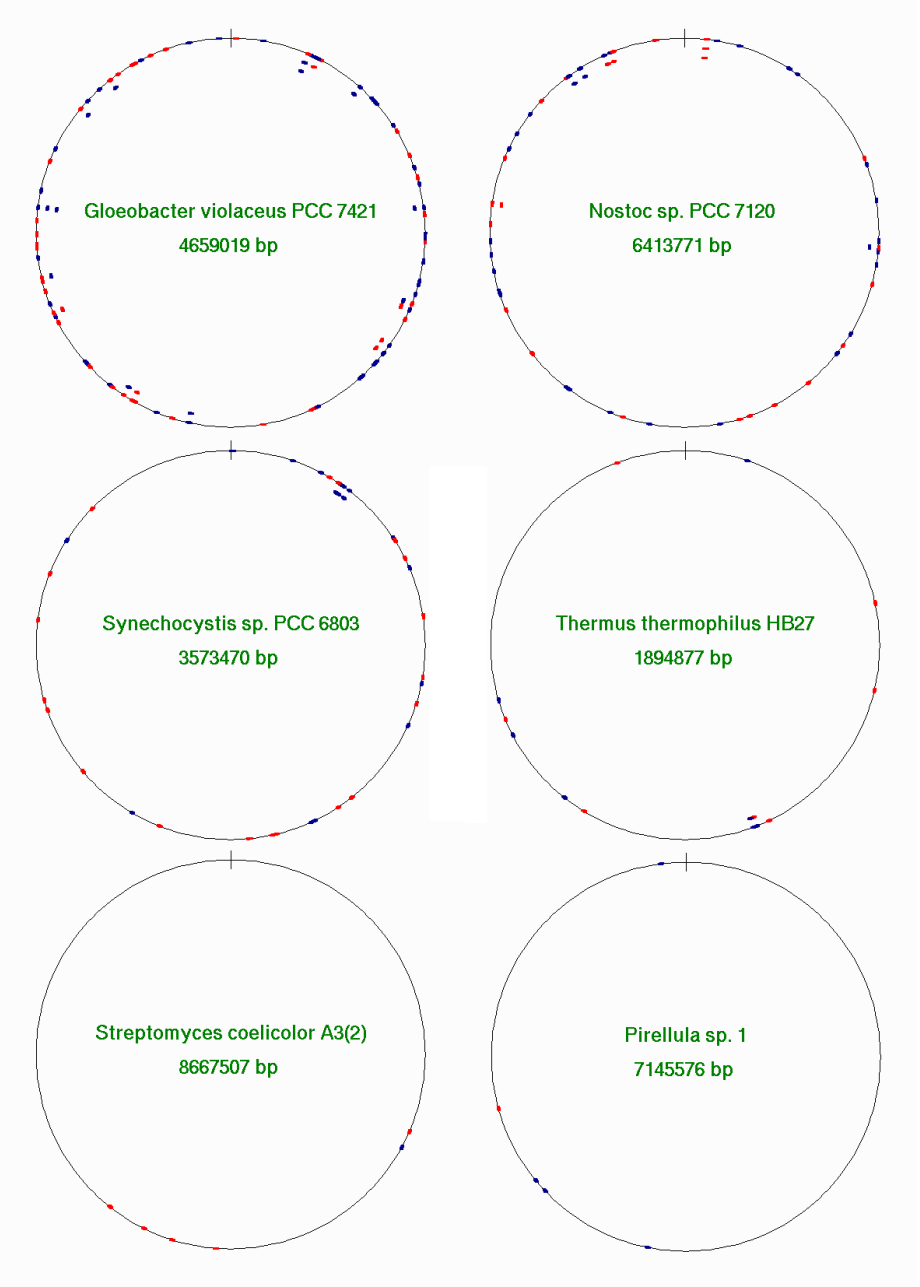
**Localization of COG4636+ family members in the chromosomes of Cyanobacteria with completed genomes. **Circular chromosome maps of genomes with at least three genes encoding COG4636+ members (indicated by dots). Genes shown in dark blue are transcribed clockwise (positive reading frame) and those in red are transcribed anticlockwise (negative reading frame). Dots plotted inside the circle indicate that more than one gene is localized in the same region of the map (1/360 of the genome length).

To identify if members of COG4636+ are encoded by any known mobile genetics elements or if they are preferentially associated with any other proteins, we analyzed the genomic neighborhood of all members of the family. Although we carefully examined annotations of predicted open reading frames (ORFs) in the range of 3000 bp upstream and downstream, we weren't able to identify any recurrent type of proteins, either with respect to the molecular or cellular function or the predicted three-dimensional fold (data not shown). Also no preference for occurrence of COG4636+ family members within or near any apparent mobile genetic elements (putative prophages etc.) was observed. Thus, insertion of the genes encoding putative COG4636+ nucleases seems virtually random. The only notable exception is a neighborhood of another member of COG4636+, suggesting tandem duplication. We identified one instance of 4 consecutively arranged genes in the genome of *C. watsonii *WH8501, all from the above-mentioned branch of 6 closely related sequences (the other two relatives are located elsewhere on the chromosome). We also found a few tandem duplications: 9 in *C. watsonii *WH8501 and 5 in *G. violaceus *PCC7421, 5 in Nostoc sp. PCC6803, 2 in *N. punctiforme *PCC73102, 2 in *A. variabilis *ATCC 29413, 2 in Synechocystis sp. PCC6803, 2 in *T. thermophilus *HB27, 1 in *T. erythraeum *IMS101 and 1 in *M. magnetotatcticum *MS-1. In general, however, tandem duplications are rare and the distribution of COG4636+ family members along the chromosomes of Cyanobacteria with completed genomes seems completely erratic (Figure 5).

## Discussion

Our results suggest that functionally uncharacterized proteins grouped together in COG4636 are a branch of the PD-(D/E)XK superfamily, which has not been identified to date due to a presence of an unusual variant of the active site, which lacks the conserved Lys residue at the typical position in the primary sequence. That the catalytic Lys can migrate in the framework of the active site of PD-(D/E)XK nucleases has been suggested earlier, based on the sequence analysis of another nuclease domain found in site-specific, non-long terminal repeat retrotransposable elements [2], but to date no molecular model was offered to suggest the alternative point for the attachment of the side chain to the protein backbone. Our sequence analysis of the COG4636+ family and the structural model of one of its members explain the problems with identification of the PD-(D/E)XK motif on the sequence level and provide a platform for further studies. Specifically, our analysis points at the most interesting members of the family, which display previously not observed variants of the PD-(D/E)XK active site. Experimental analyses of these proteins and determination of the role of individual amino acids in the evolutionary context may help to better understand the plasticity of the PD-(D/E)XK active site and may settle down the controversy in the field of nucleases regarding the mechanism(s) of the reaction.

Phylogenomic analyses show that putative nucleases grouped in the COG4636+ family are exceptionally abundant in genomes of certain Cyanobacteria, but absent in others. They are typically abundant in the sequenced genomes of freshwater species, but scarce in the genomes of marine species, with the exception of *C. watsonii *WH 8501, which was isolated from tropical waters of the Western Atlantic and Pacific oceans. It is remarkable that members of COG4636+ are almost absent from the genomes of *Synechococcus *and *Prochlorococcus *species thriving in the Sargasso sea, as well as in the environmental samples isolated from that region. On the other hand, in *G. violaceus *PCC 7421 they comprise over 2% of all protein-encoding genes. This phylogenetic distribution resembles that of mobile genetic elements such as introns or insertion sequences (reviews: [41,42]) and suggests that the contemporary COG4636+ family originated from a few predecessors that underwent extensive horizontal gene transfer and massive proliferation in certain genomes. Monophyly of COG4636+ sequences in non-Cyanobacterial species strongly suggests that proliferation occurred in each of these species independently, following a single event of colonization by horizontal transfer from a Cyanobacterium (or in the case of *T. thermophilus *– three independent successful colonizations).

We hypothesize that the mechanism by which these putative nucleases induce their proliferation in a genome is similar to that displayed by homing nucleases and restriction enzymes [43], namely to incise the DNA by introducing nicks or double-strand breaks, which stimulates recombination and may lead to tandem duplications and a variety of genomic rearrangements [44-47]. Frequent cleavage of the genomic DNA would be lethal for the cell, therefore if members of COG4636+ are indeed active as nucleases, then they should target rare sequences (in a manner similar to homing endonucleases; review: [48]) or unusual structures in the DNA (similarly to the structure-specific Holliday junction resolvases), or their activity would have to be somehow regulated (inhibited) by interactions with other proteins or cellular processes (for instance by DNA modification). There are known examples of Holliday junction resolvases carried on defective lambdoid prophages [49]. Unfortunately, analysis of the genomic neighborhood shows no preferred association of COG4636+ members with any mobile genetic elements or particular gene families that could give us hints about the cellular processes they could be part of or suggest how their predicted nuclease activity could be inhibited or regulated. Especially, we found no correlation with the presence of known or putative methyltransferases. This suggests that despite sharing the common PD-(D/E)XK fold with REases, COG4636+ members are unlikely to serve as parts of restriction-modification systems, which are known to be abundant in Cyanobacteria [50,51]. It must be noted, however, that multiple solitary DNA methyltransferases were reported in *Anabaena *PCC 7120 [51], and these enzymes could potentially provide protection against the cleavage of the chromosomal DNA by at least some of the COG4636+ members found in this organism.

One possibility is that COG4636+ members serve as a part of the restriction barrier, similarly to the unrelated NucA family of extracellular nucleases found in Cyanobacteria, e.g. *Anabaena *sp. PCC 7120 [52] and *Microcystis *sp. [53]. They could also fulfill a role in maintenance of the identity of the species by controlling the flow of incoming DNA, as recently suggested for restriction-modification systems [54]. From the genomic analyses it appears, however, that the primary function of COG4636+ members is to spread and multiply, and their cellular roles may be merely side-effects of this selfish expansion. It is very likely that their nuclease activity is recombinogenic and may increase the frequency of genomic rearrangements. Moreover, the multiplication of closely related COG4636+ members in certain genomes leads to an abundance of dispersed related DNA sequences, which by themselves may increase the frequency of genome rearrangements by homologous recombination. It was suggested that in the marine Cyanobacteria the factors that increase the genome plasticity might not be promoted by natural selection due to the homeostatic environment of the open ocean [55]. Conversely, the unstable environment of fresh waters might promote the spreading of factors that destabilize the genome by increasing the frequency of recombination and thereby increase the diversity of the population. This is in good agreement with our finding of prevalence of COG4636+ members in Cyanobacteria that thrive in fresh waters and their paucity in marine species (with the exception of *C. watsonii *WH 8501). Summarizing, it is plausible that members of COG4636+ fulfill an important role in the genome dynamics of Cyanobacteria and other species they colonize. We hope that our predictive study will facilitate experimental determination of the molecular and cellular function of members of this intriguing protein family.

## Methods

### Sequence analysis

Searches of the non-redundant (nr) database were carried out at the NCBI using PSI-BLAST [56] with default parameters, using different sequences from COG4636 as queries. Significantly similar sequences were retrieved from all searches and pooled together. Identical sequences from the same organism were removed. A multiple sequence alignment was generated using MUSCLE [57] with default parameters and subsequently adjusted manually, based on the analysis results of secondary structure prediction (see below), to ensure that no unwarranted gaps are introduced within α-helices and β-strands. Phylogenetic inference was carried out using the reliable central section of the multiple sequence alignment. The matrix of pairwise distances was calculated from sequences according to the JTT model [58] with gaps treated as missing data. The neighbor-joining (NJ) tree was inferred according to the method of Saitou and Nei [59].

### Phylogenomic analysis

The Eutils module from the Biopython package was used as an interface to access remotely the NCBI databases [60]. The Gene Identification numbers of proteins included in the final multiple alignment sequences were used to identify the corresponding GenPept entries, which were downloaded into a local Barkeley database using an in-house developed parser based on the SAX package . The "coded_by" field from each GenPept file was used to identify the corresponding DNA sequence, which were also downloaded into the database. The sequence in the range of 3000 bp upstream or downstream from the region encoding a COG4636+ member were scanned for the presence of annotated Open Reading Frames (ORFs). Initially, the functional categorization of these ORFs was carried out based on the automatic assignment into the PFAM and COG families. In the absence of any recurrent function, the annotations of all ORFs were carefully re-analyzed visually and in uncertain cases, additional searches against the CDD database were carried out [61]. The distribution of COG4636+ members on the chromosome maps was visualized using a program developed in-house specifically for that purpose.

### Protein structure prediction

Secondary structure prediction and tertiary fold-recognition was carried out via the GeneSilico meta-server gateway at [33]. Secondary structure was predicted using PSIPRED [62], PROFsec [63], PROF [64], SABLE [65], JNET [66], JUFO [67], and SAM-T02 [68]. Solvent accessibility for the individual residues was predicted with SABLE [65] and JPRED [66]. The fold-recognition analysis (attempt to match the query sequence to known protein structures) was carried out using FFAS03 [69], SAM-T02 [68], 3DPSSM [70], BIOINBGU [71], FUGUE [72], mGENTHREADER [73], and SPARKS [74]. Fold-recognition alignments reported by these methods were compared, evaluated, and ranked by the Pcons server [35].

### Homology modeling

Fold-recognition alignments to the structures of selected templates were used as a starting point for homology modeling using the "FRankenstein's Monster" approach [34], comprising cycles of model building, evaluation, realignment in poorly scored regions and merging of best scoring fragments. The positions of predicted catalytic residues and secondary structure elements were used as spatial restraints. Briefly, preliminary models were generated based on the alignments to various template structures returned by the FR servers. The sequence-structure fit in these models was assessed using VERIFY3D [75] and visualized using the COLORADO3D server [76]. The most common and best-scoring fragments were merged to produce a hybrid model, in which the sequence-structure was re-evaluated. In the poorly scoring fragments the alignment was locally modified by shifting the sequences within the limits of predicted secondary structures and a next generation of models corresponding to different alignments was generated. The cycles of evaluation of models, generation of hybrids and local re-alignment in problematic regions continued until the global VERIFY3D score could not be improved. Regions, which could not be modeled because of the lack of the appropriate template structure, were added "de novo" using the fragment insertion method ROSETTA [37].

## Note added in Proof

**Figure 6:**
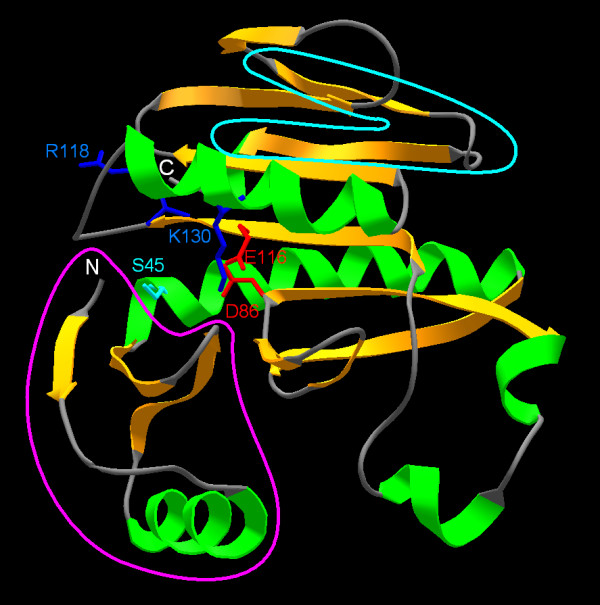
**The crystal structure of Tt1808 (1wdj in PDB). **Tt1808 is shown in the same orientation and is colored and labeled in the same way as the homology model of all3650 on Figure 3. Two regions of differences between Tt1808 and the model of all3650 are indicated: the N-terminal subdomain has a similar fold, but different orientation (magenta line) and the C-terminal region folds as a β-harpin (cyan line) rather than as an α-helix.

After submission of this manuscript, a crystal structure of one of the COG4636+ members was released in the Protein Data Bank (code 1wdj; Idaka, M., Wada, T., Murayama, K., Terada, T., Kuramitsu, S., Shirouzu, M., Yokoyama, S.: Crystal Structure of Tt1808 from *Thermus thermophilus *Hb8 To be Published). Our analysis of the Tt1808 structure and its comparison with the model of all3650 confirms our predictions. Tt1808 does indeed exhibit the PD-(D/E)xK fold: the DALI [77] search of the the Protein Data Bank (PDB) database with 1wdj revealed that its 8 closest structural matches with Z-scores in a range of 5.3-3.7 are members of the PD-(D/E)xK superfamily, including the Holliday junction resolvases we used as templates to model the all365 protein. Analysis of the Tt1808 structure (Figure 6) reveals that we correctly predicted the topology of the catalytic domain in all365. We only mispredicted an α-helix in the C-terminus of all365; in Tt1808 this element is replaced by a β-hairpin. We have also successfully modeled the structure of the N-terminal subdomain but failed to predict the interaction between this part and two loops of the catalytic domain (compare Figure 3 and Figure 6). It is important to note that these errors concern regions that do not influence any of our functional interpretations based on the all3650 model. Most importantly, the identity of presumed catalytic residues of all365 was predicted correctly, including the postulated unusual position of the Lys residue (in our model of all365 the side chain of K127 has a different orientation than K130 in Tt1808, but such details are irrelevant to our functional interpretations). It is interesting to note that Tt1808 has the S-PD-EXR-K variant of the active site, and that the side chain of the R118 residue, which replaced the "classical" catalytic Lys, points away from other catalytic residues, on the opposite side of the loop between the "EXR" and "K" elements. Summarizing, we correctly predicted all functionally important features of the COG4636+ family, including the membership in the PD-(D/E)xK superfamily of nucleases, the three-dimensional fold, the putative catalytic residues, and the unusual configuration of the active site.

## List of abbreviations

aa, amino acid(s); bp, base pair(s); nt, nucleotide; e, expectation; REase, restriction endonuclease; ORF, product of an open reading frame,

## Authors' contributions

MF carried out all sequence analyses and structure predictions using fold-recognition methods and ROSETTA. JMB built the homology model, analyzed spatial vs. sequential conservation of the putative active site, and wrote the manuscript. Both authors have read and accepted the final version of the manuscript.

**Table 1:** Distribution of COG4636+ family members among different bacteria.

organism / genome	phylum	habitat	data source	COG4636+ members	
				
				total	disrupted
Gloeobacter violaceus PCC 7421	Cyanobacteria	calcareous rock	C	95	1
*Nostoc punctiforme *PCC 73102	Cyanobacteria	cycad (endosymbiont)	WGS	71	7
*Crocosphaera watsonii *WH 8501	Cyanobacteria	marine water	WGS	62	1
*Nostoc *sp. PCC 7120	Cyanobacteria	fresh water	C	58	1
*Anabaena variabilis *ATCC 29413	Cyanobacteria	fresh water	WGS	45	5
*Synechocystis *sp. PCC 73102	Cyanobacteria	fresh water	C	36	1
*Thermus thermophilus *HB27	Deinococcus-Thermus	thermal environment	C	14	-
*Trichodesmium erythraeum *IMS101	Cyanobacteria	marine water	WGS	10	3
*Desulfitobacterium hafniense *DCB-2	Firmicutes	sewage sludge	WGS	8	-
*Chloroflexus aurantiacus*	Chloroflexi	fresh water (hot springs)	WGS	7	-
*Streptomyces coelicolor *A3(2)	Actinobacteria	soil	C	6	-
*Rhodopirellula baltica *SH 1	Planctomycetes	marine water	C	5	1
*Moorella thermoacetica *ATCC 29413	Firmicutes	fresh water (ponds)	WGS	3	-
*Deinococcus radiodurans *R1	Deinococcus-Thermus	unknown	C	3	-
*Magnetospirillum magnetotacticum *MS-1	Proteobacteria	fresh water (ponds)	WGS	2	1
*Synechococcus elongatus *PCC 73102	Cyanobacteria	fresh water	WGS	2	-
*Aquifex aeolicus *VF5	Aquificae	fresh water (hot springs)	C	2	-
*Kineococcus radiotolerans *SRS30216	Actinobacteria	unknown (isolated from radioactive work area)	WGS	2	-
*Caulobacter crescentus *CB15	Proteobacteria	fresh water	C	1	-
*Thermosynechococcus elongatus *BP-1	Cyanobacteria	fresh water (hot springs)	C	1	-
*Synechococcus *sp. PCC 73102	Cyanobacteria	brackish (euryhaline) and/or marine water	UGS	1	-
*Microcystis aeruginosa*	Cyanobacteria	fresh water (lakes, ponds and rivers)	NR	1	-
Prochlorococcus marinus str. MIT9313	Cyanobacteria	marine water	C	-	-
*Prochlorococcus marinus *subsp. marinus CCMP1375	Cyanobacteria	marine water	C	-	-
*Prochlorococcus marinus *subsp. pastoris CCMP1986	Cyanobacteria	marine water	C	-	-
*Synechococcus *sp. WH 8102	Cyanobacteria	marine water	C	-	-

C – Completed genomic sequence, WGS – Whole Genome Shotgun, UGS – Unfinished Genomic Sequence, NR – non-redundant database (NCBI). ORFs were regarded as "disrupted" if they bear frameshift mutations or stop codons.

## Supplementary Material

Additional File 1 The additional data file all3650.pdb contains the coordinates of the original all3650 model (obtained before the Tt1808 structure was published) in the PDB format. Click here for file
